# Supplementary material for: ATP binds and inhibits the neurodegeneration-associated fibrillization of the FUS RRM domain
Source: Commun Biol. 2019 Jun 20;2:223. doi: 10.1038/s42003-019-0463-x (PMC6586847; doi:10.1038/s42003-019-0463-x)
Supplement: Supplementary file 1 — Supplementary Information [file 42003_2019_463_MOESM1_ESM.pdf]

Supplementary Figure 1

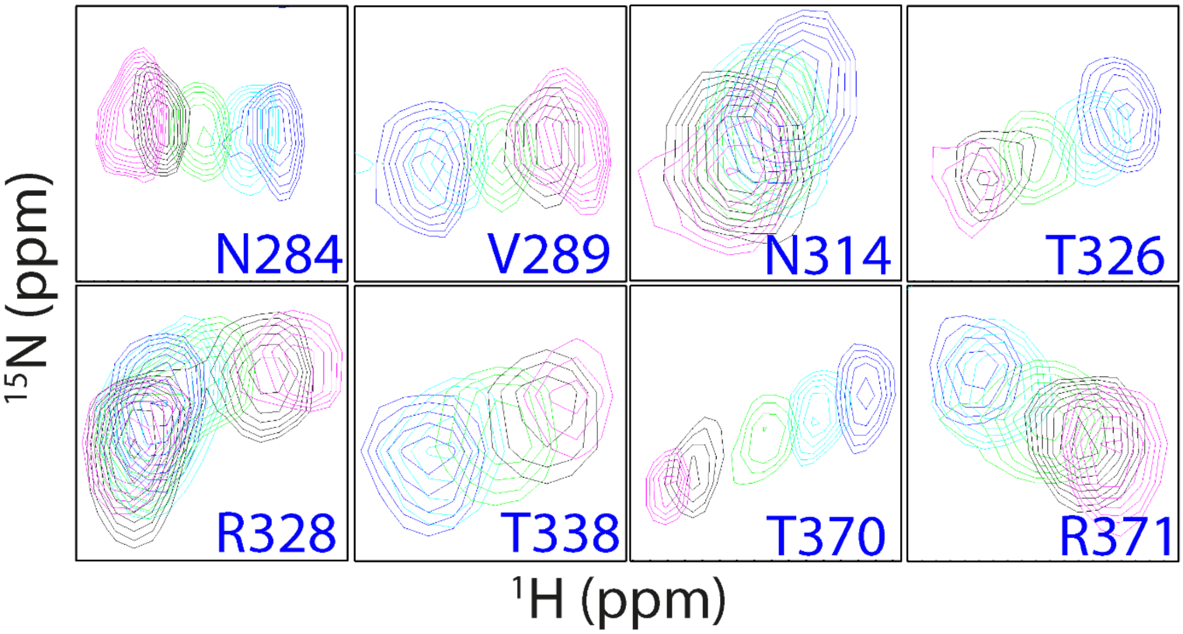

Supplementary Figure 1. NMR HSQC titrations of FUS RRM domain with ATP. Traces of eight HSQC peaks of FUS RRM domain, which have large shifts but are not severely overlapped with other peaks. For clarity, only peaks at five ATP concentrations are shown: in the free state (blue); in the presence of ATP at 4 mM (cyan); 10 mM (green); 22 mM (black); 40 mM (pink).

Supplementary Figure 2

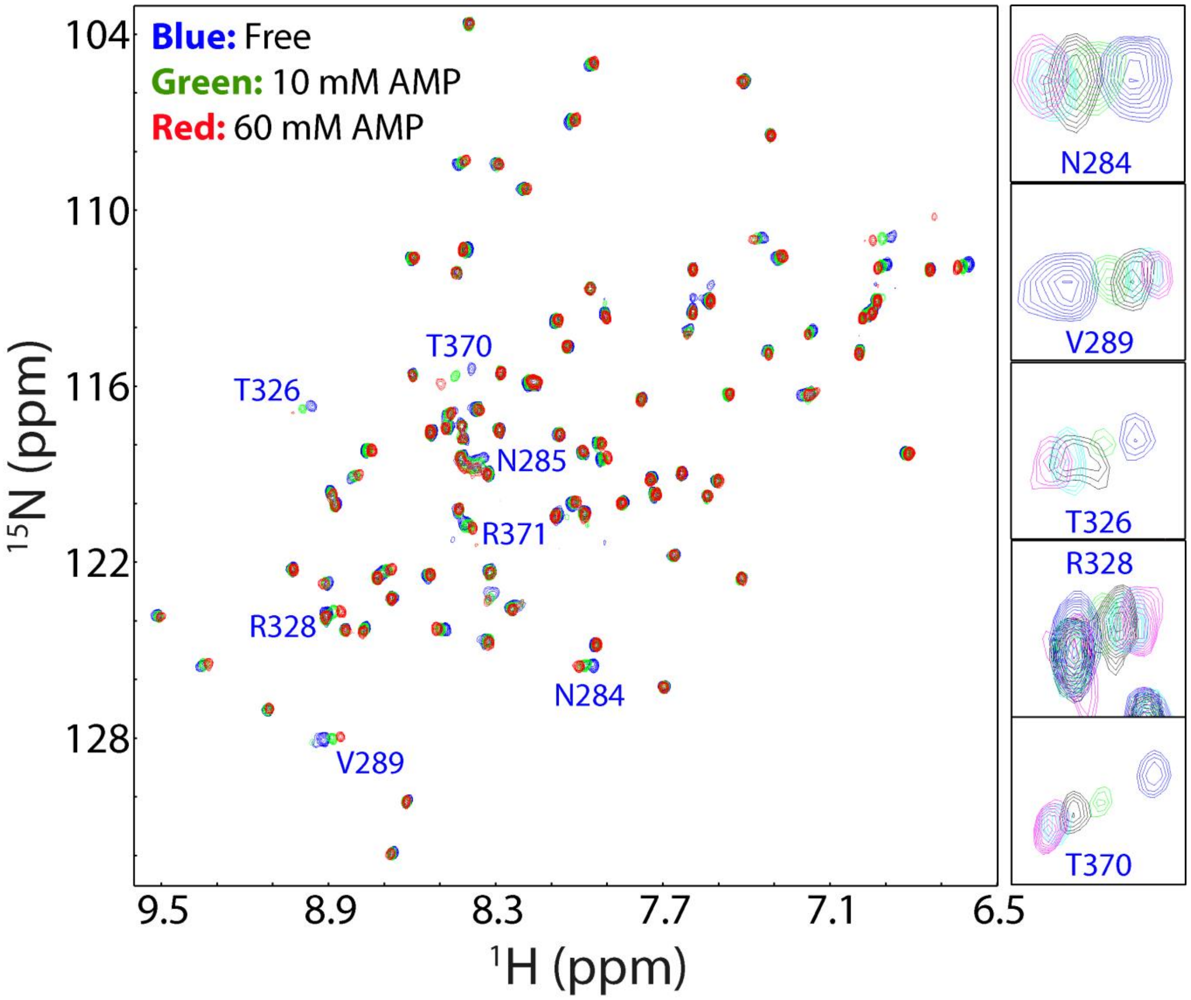

Supplementary Figure 2. NMR HSQC titrations of FUS RRM domain with AMP. ^{1}H-^{15}N NMR HSQC spectra of the ^{15}N-labeled FUS RRM domain in the absence and in the presence of AMP at different concentrations, as well as traces of six HSQC peaks, which have largely shifts but are not severely overlapped with other peaks. For clarity, only peaks at five AMP concentrations are shown: in the free state (blue); in the presence of AMP at 10 mM (green); 30 mM (black); 50 mM (cyan); 60 mM (pink).

Supplementary Figure 3

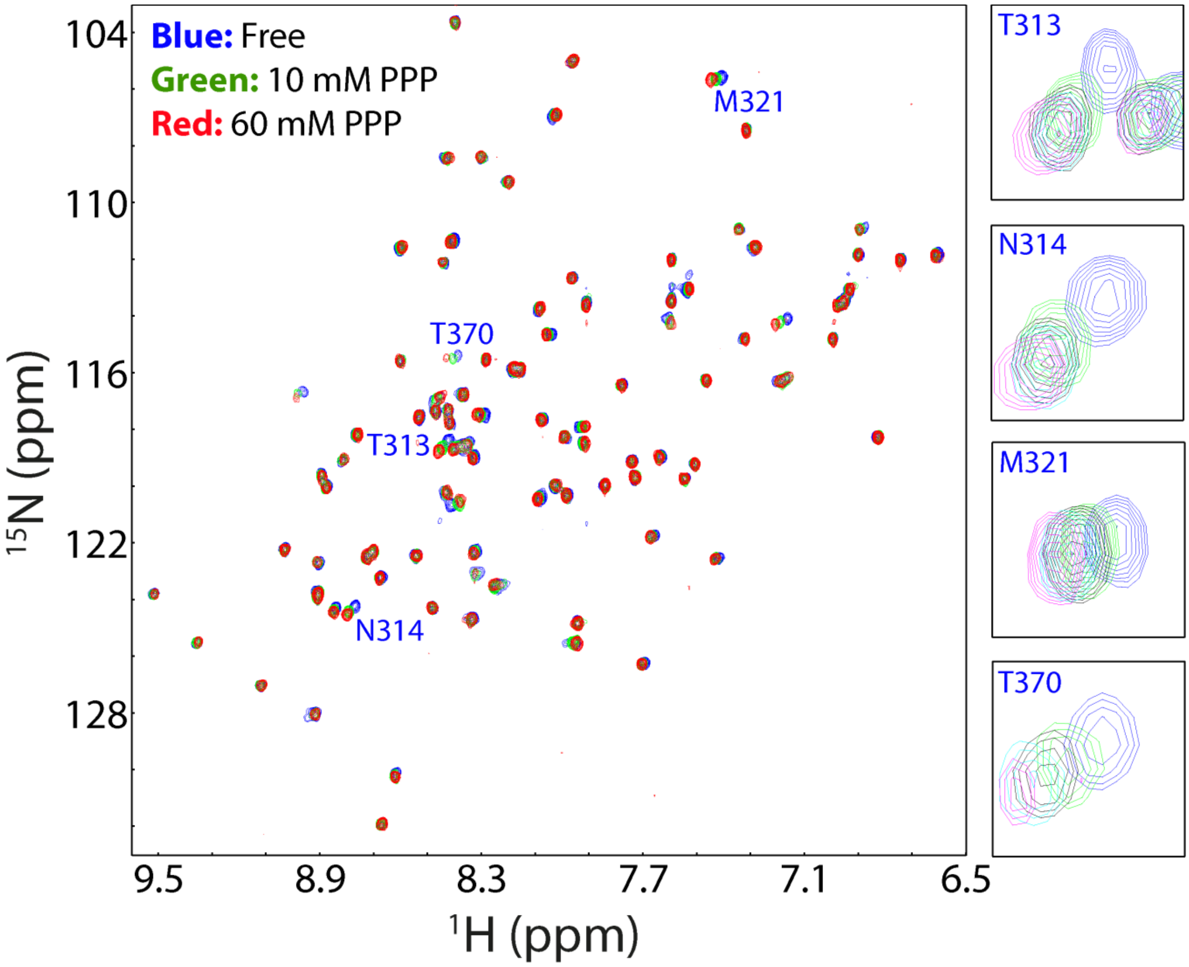

Supplementary Figure 3. NMR HSQC titrations of FUS RRM domain with PPP.

^{1}H-^{15}N NMR HSQC spectra of the ^{15}N-labeled FUS RRM domain in the absence and in the presence of PPP at different concentrations, as well as traces of four HSQC peaks, which have large shifts. For clarity, only peaks at five PPP concentrations are shown: in the free state (blue); in the presence of PPP at 10 mM (green); 30 mM (black); 50 mM (cyan); 60 mM (pink).

Supplementary Figure 4

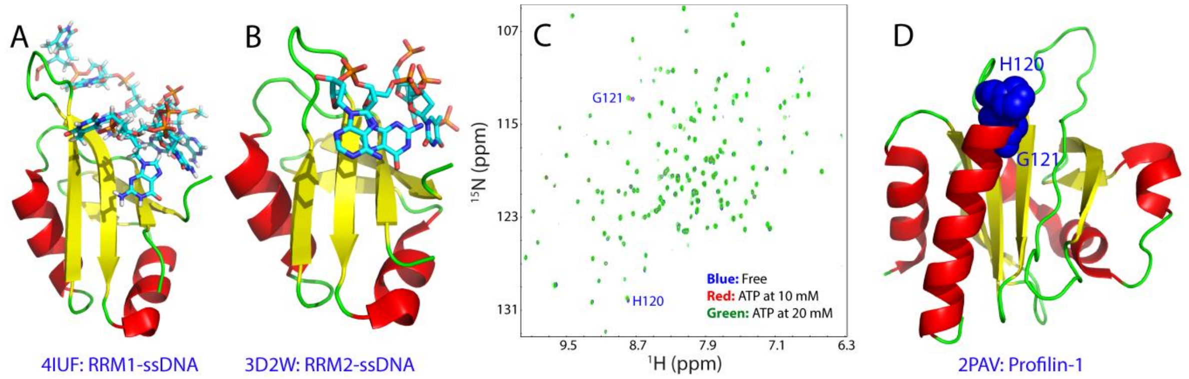

Supplementary Figure 4. ATP has no large binding to profilin-1.
(A)-(B) Crystal structures of TDP-43 RRM1 (Ref. 24) and RRM2 (Ref. 25) in complex with ssDNA. (C) ^{1}H-^{15}N HSQC spectra of the human profilin-1 in the absence and in the presence of ATP at different concentrations. The assignments of two residues (His120 and Gly121) with shifted HSQC peaks are labeled. (D) Crystal structure of the human profilin-1 (Ref. 27) with two residues His120 and Gly121 displayed in blue spheres.

Supplementary Figure 5

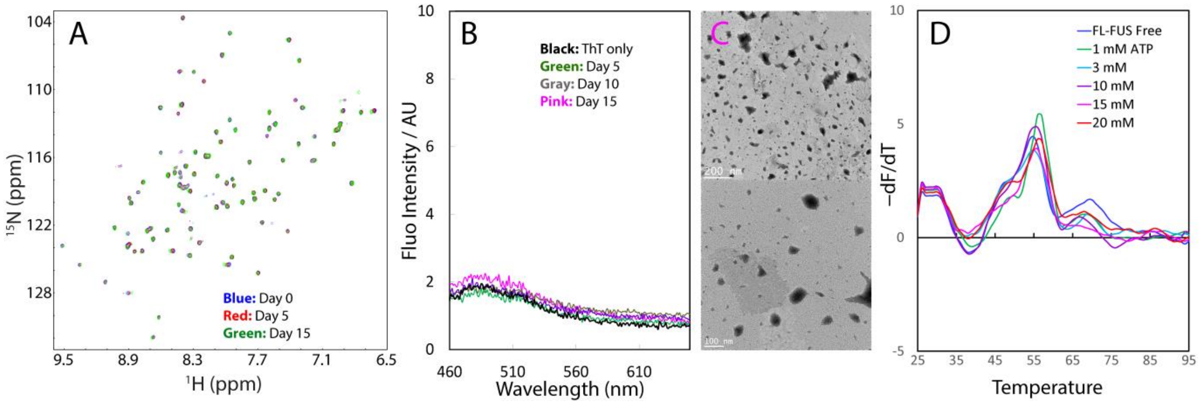

Supplementary Figure 5. Effects of ATP on the fibrillation and stability.
(A) ^{1}H-^{15}N NMR HSQC spectra of the ^{15}N-labeled FUS RRM domain in the presence of 3 mM ATP at different time points of incubation. (B) Emission spectra of the ThT-binding induced fluorescence for FUS RRM domain at different time points of incubation, which have the typical emission maximum at ~486 nm. (C) EM images of FUS RRM domain sample after 15 days of incubation. (D) DSF melting curves of thermal unfolding of the full-length FUS in the presence of ATP at different concentrations by plotting the first derivative of the fluorescence emission as a function of temperature (−dF/dT).
